# Impaired Muscle Regeneration in Ob/ob and Db/db Mice

**DOI:** 10.1100/tsw.2011.137

**Published:** 2011-07-28

**Authors:** Mai-Huong Nguyen, Ming Cheng, Timothy J. Koh

**Affiliations:** Department of Kinesiology and Nutrition, University of Illinois at Chicago, USA

**Keywords:** skeletal muscle injury, tissue repair, obesity, inflammation, leptin

## Abstract

In obesity and type 2 diabetes, efficient skeletal muscle repair following injury may be required, not only for restoring muscle structure and function, but also for maintaining exercise capacity and insulin sensitivity. The hypothesis of this study was that muscle regeneration would be impaired in ob/ob and db/db mice, which are common mouse models of obesity and type 2 diabetes. Muscle injury was produced by cardiotoxin injection, and regeneration was assessed by morphological and immunostaining techniques. Muscle regeneration was delayed in ob/ob and db/db mice, but not in a less severe model of insulin resistance – feeding a high-fat diet to wild-type mice. Angiogenesis, cell proliferation, and myoblast accumulation were also impaired in ob/ob and db/db mice, but not the high-fat diet mice. The impairments in muscle regeneration were associated with impaired macrophage accumulation; macrophages have been shown previously to be required for efficient muscle regeneration. Impaired regeneration in ob/ob and db/db mice could be due partly to the lack of leptin signaling, since leptin is expressed both in damaged muscle and in cultured muscle cells. In summary, impaired muscle regeneration in ob/ob and db/db mice was associated with reduced macrophage accumulation, angiogenesis, and myoblast activity, and could have implications for insulin sensitivity in the skeletal muscle of obese and type 2 diabetic patients.

## INTRODUCTION

Obesity and type 2 diabetes are associated with impairments in skeletal muscle function, including insulin resistance, ischemia, impaired oxidative capacity, and decreased muscle force production per unit area[1,2,3,4]. Proper muscle function is required for regular physical activity, which is a cornerstone strategy for losing weight, improving insulin sensitivity, and reducing the complications associated with obesity and diabetes[5,6]. Consequently, impairments in muscle function associated with type 2 diabetes can limit the ability of patients to undertake therapeutic exercise regimens. In addition, the skeletal muscle can be injured by vigorous physical activity, peripheral ischemia, or trauma. Such muscle damage itself can induce an insulin-resistant state[7,8]. Thus, efficient muscle repair following injury may be required, not only for restoring muscle structure and function, but also for maintaining exercise capacity and insulin sensitivity.

Ob/ob and db/db mice are obese and diabetic, and are widely used to study the pathophysiology of type 2 diabetes. These mice have mutations in the genes coding for leptin and the leptin receptor, respectively; leptin is a cytokine that has received much attention for its role as a regulator of energy balance[9]. Ob/ob and db/db mice are hyperglycemic, hyperinsulinemic, and insulin resistant, and demonstrate impaired tissue inflammatory responses in the cornea, skin, peritoneum, and gut[10,11,12,13]. A number of studies have shown that skin wound healing is impaired in ob/ob and db/db mice, which is associated with a dysregulated inflammatory response[10,14]. However, a review of the literature revealed no studies on muscle inflammation and repair in these obese and diabetic mice.

Similar to other tissues, skeletal muscle regeneration involves overlapping phases of inflammation, new tissue formation, and remodeling[15]. The inflammatory phase involves accumulation of neutrophils and macrophages that debride damaged tissue and stimulate the healing response. In muscle, the tissue formation phase involves the generation of new muscle fibers, formed by the proliferation and fusion of resident muscle precursor cells called satellite cells. Finally, the remodeling phase involves growth of newly formed fibers and remodeling of the extracellular matrix. Recent data indicate that macrophages are likely involved in stimulating satellite cell activity through production of soluble factors[16,17] and are required for efficient muscle healing[17,18,19]. Since the function of both macrophages and muscle cells may be impaired by a lack of leptin signaling and its downstream consequences, each phase of muscle regeneration could be impaired in ob/ob and db/db mice.

The hypothesis of this study was that muscle regeneration would be impaired in ob/ob and db/db mice. Our data indicate that following cardiotoxin-induced injury, macrophage accumulation is impaired in ob/ob and db/db mice, which is associated with reduced angiogenesis, cell proliferation, myoblast accumulation, and muscle regeneration.

## MATERIALS AND METHODS

- **Mice** — Leptin-deficient (ob/ob) and leptin receptor–mutant (db/db) mice on a C57Bl/6 background, along with C57Bl/6 mice, were obtained from Jackson Laboratories. Ob/ob and db/db mice are obese and diabetic, and are widely used as mouse models of type 2 diabetes. In addition, a group of wild-type (WT) C57Bl/6 mice were fed a high-fat diet (HFD, 60% kcal from fat) starting at 4 weeks of age and continuing for 12 weeks, while all other mice received normal chow (NC, 6% kcal from fat). This HFD protocol is widely used to induce obesity and insulin resistance[20], which is less severe than in ob/ob and db/db mice. Experiments were performed on 14- to 16-week-old mice. All experimental procedures were approved by the Animal Care Committee at the University of Illinois at Chicago.
- **Injury Model** — Mice were anesthetized with an intraperitoneal injection of ketamine (100 mg/kg) and xylazine (5 mg/kg), and their hindlimbs were shaved and then cleaned with betadine and alcohol swab. Extensor digitorum longus (EDL) muscles were injured using cardiotoxin as previously described[18,21]. Briefly, cardiotoxin (10 μM; EMD Chemicals) was administered with two intramuscular injections per muscle to ensure distribution throughout the muscle. At 1, 3, 5, or 10 days following injury, mice were euthanized by cervical dislocation while under anesthesia, and EDL muscles were harvested and mounted in tissue-freezing medium and frozen in isopentane chilled with dry ice for histological analysis, or snap frozen in liquid nitrogen for gene expression analysis.
- **Morphology** — Muscle cross-sections were stained with hematoxylin and eosin, and quantitative analysis was performed on five representative images of each muscle section obtained using a 40X objective with a Nikon Labphot-2 microscope and SPOT camera and software. For each field, fibers were classified as normal, damaged, or regenerating as described[18,21]. Regenerating fibers were identified as those containing centrally located nuclei without evidence of damage. The number and area of each type of fiber were recorded. The damaged area was then estimated in each muscle section by subtracting the summed area of normal and regenerating fibers from the total area of each field.

- **Immunofluorescence** — Cell proliferation was assessed in the muscle cryosections using the incorporation of BrdU into the cell nuclei. One hour prior to euthanasia, mice were injected with 30 mg/kg BrdU. Cross-sections were cut from the mid-belly of each EDL muscle (10-µm thickness), fixed in cold acetone, washed in PBS, and incubated in 2 N HCl to denature DNA. Sections were neutralized in basic PBS and then incubated in a blocking buffer containing 0.2% gelatin and 3% BSA. Proliferating cells were labeled with a BrdU antibody (Roche) for 1 h and subsequently incubated with FITC antimouse secondary antibody (Jackson ImmunoResearch). Alternatively, sections were stained for the myogenic transcription factor MyoD. These sections were fixed in cold acetone, incubated with blocking buffer, and then incubated with primary antibody (Santa Cruz Biotechnology) overnight. Sections were subsequently incubated with TRITC antirabbit secondary antibody (Jackson ImmunoResearch) and then mounted in medium containing DAPI. The number of DAPI-positive nuclei that were also MyoD- or BrdU-positive was counted in three fields observed using a 40X objective with a Nikon Labphot-2 microscope and SPOT digital camera and software (Diagnostic Instruments) for two sections per muscle and normalized to the volume of muscle sampled.
- **Immunohistochemistry** — Immunohistochemical analysis was performed on cryosections essentially as described[18,21]. Sections were air dried, fixed in cold acetone, washed with PBS, quenched with 0.3% hydrogen peroxide, and washed with PBS. Sections were blocked with buffer containing 3% BSA and then incubated with F4/80 antibody to label macrophages (1:50, Serotec), or Ly6G antibody to label neutrophils (1:100, BD Pharmingen). Sections were then washed with PBS and incubated with biotinylated antirat secondary antibody (1:200, Vector Laboratories). After washing with PBS, sections were incubated with avidin D-horseradish peroxidase (1:1000) and developed with a 3-amino-9-ethylcarbazole kit (Vector Laboratories). The number of labeled cells was counted in two sections per muscle using a 20X objective (Labphot-2, Nikon), and then normalized to the volume of the muscle section = section thickness × area, the latter measured using a 2X objective (SPOT software, Diagnostic Instruments).
- **Muscle Cell Cultures** — C2C12 myoblasts (ATCC), a cell line derived from mouse skeletal muscle satellite cells, were plated at $1 \times 10^5$ cells/well in six-well plates in growth medium -- Dulbecco's Modified Eagles Medium (DMEM) supplemented with 10% fetal bovine serum and 1% penicillin/streptomycin (Gibco) at 37°C and 5% $CO_2$. Myoblasts were collected at 70 and 100% confluence. For some wells, when cells reached confluence, the medium was changed to differentiation medium (DMEM + 2% horse serum) and resulting myotubes were collected at 2 and 4 days after the induction of differentiation.
- **RT-PCR** — Total RNA was isolated from injured muscle tissue and cultured C2C12 muscle cells using the RNeasy kit (Qiagen) according to the manufacturer's instructions. RNA quantity was determined by UV absorption at 260 nm, and quality was verified by the 260/280 nm ratio and formaldehyde-agarose gel electrophoresis. RNA (2 µg) was reverse transcribed using the Thermoscript RT-PCR kit (Invitrogen) and PCR was performed with primers for leptin (F: CCTGTGGCTTTGGTCCTATCTG, R: AGGCAGGCTGGTGAGGACCTG) and GAPDH (F: TCTGACGTGCCGCCTGGAGA, R: GGGGTGGGTGGTCCAGGGTT). Cycling conditions were optimized to ensure that each target was within its linear range. Images of ethidium bromide–stained gels were analyzed by densitometry and expression of each target gene was normalized to that of GAPDH.
- **Statistics** — Values are reported as means ± SE. Data were compared across time points and/or mouse groups using analysis of variance (ANOVA). The Student-Newman-Keuls test was used for posthoc analysis of ANOVA tests found to be statistically significant. The 0.05 level was taken to indicate statistical significance.

## RESULTS

To determine whether muscle regeneration is impaired in ob/ob and db/db mice, or in WT mice fed a HFD compared with WT mice fed NC, we injured muscles in these mice using cardiotoxin. As expected, muscle regeneration was robust in WT mice fed NC, with a large number of regenerating muscle fibers formed and nearly 80% of the muscle cross-sectional area regenerated by 10 days postinjury (Fig. 1). In contrast, ob/ob and db/db mice both showed delayed muscle regeneration, with significantly fewer regenerating fibers formed at 5 days postinjury that approached WT levels by 10 days (Fig. 1). The total muscle area regenerated was still significantly decreased at 10 days postinjury in db/db mice. Interestingly, muscle regeneration was not different in WT mice fed the HFD for 12 weeks compared to mice fed the normal diet, indicating that the obesity and insulin resistance associated with this feeding protocol[20] is not sufficient to impair muscle regeneration. WT mice fed the HFD exhibited larger body mass (40.7 ± 1.5 g vs. 26.3 ± 0.9 g) and higher fasting blood glucose (192.3 ± 13.2 mg/dl vs. 144.4 ± 8.4 mg/dl) compared with WT mice fed NC, providing evidence that the HFD had the desired effect of inducing obesity and hyperglycemia.

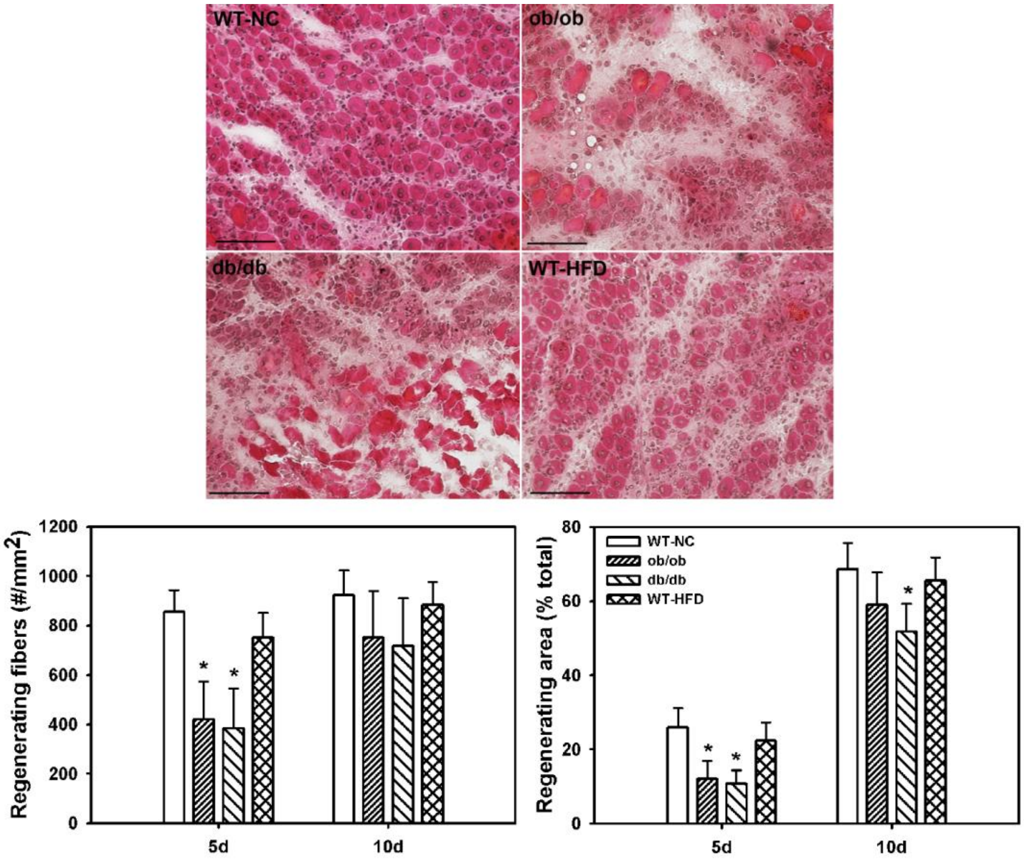

**FIGURE 1.** Muscle regeneration is impaired in ob/ob and db/db mice. Injured muscles collected from WT mice fed NC, ob/ob mice, db/db mice, and WT mice fed HFD. Top: cryosections of muscles collected on day 5 postinjury and stained with hematoxylin and eosin for morphological analysis. Note the relative lack of central-nucleated (regenerating) muscle fibers in ob/ob and db/db mice. Scale bar = 0.1 mm. Bottom: summary data for number of regenerating muscle fibers/$mm^2$ and total regenerating area as a percentage of the total muscle cross-sectional area on days 5 and 10 postinjury. Bars are means ± standard error, n = 4–6 per time point. * = Mean value significantly smaller than that for WT-NC mice; $p < 0.05$.

New muscle fibers are formed during muscle regeneration by activation, proliferation, and fusion of resident stem cells called satellite cells[15,22]. BrdU was injected into mice 1 h prior to muscle harvest to label proliferating cell nuclei, and MyoD labeling was performed to assess the net effect of satellite cell activation and proliferation. In WT mice fed NC, cell proliferation and MyoD+ cell accumulation was robust at 5 days postinjury (Fig. 2), the time point at which we have previously observed a peak in these assays[23]. In ob/ob and db/db mice, the number of Brdu+ and of MyoD+ cells was significantly reduced (Fig. 2), indicating impaired cell proliferation and myoblast accumulation in these obese and diabetic mice. Again, WT mice fed the HFD diet did not differ in these measurements of muscle regeneration compared with WT mice fed NC.

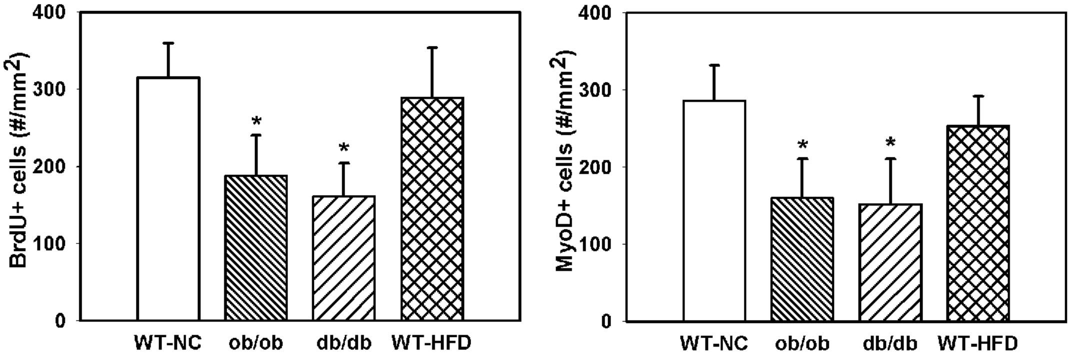

**FIGURE 2.** Cell proliferation and myoblast accumulation following muscle injury is impaired in ob/ob and db/db mice. Injured muscles collected from WT mice fed NC, ob/ob mice, db/db mice, and WT mice fed HFD. Left: summary data for BrdU incorporation into newly synthesized DNA on day 5 postinjury, expressed as the number of nuclei staining positive for BrdU/mm$^2$ muscle area. Right: summary data for number of nuclei staining positive for MyoD on day 5 postinjury, expressed per mm$^2$ muscle area. Bars are means ± standard error, n = 4–6 per time point. *= Mean value significantly smaller than that for WT-NC mice; $p < 0.05$.

Tissue damage in obese and type 2 diabetic mice has been previously associated with dysregulated inflammatory response[10,12,14]. Following muscle injury, neutrophils and then macrophages invade damaged muscle and macrophages at least are required for efficient muscle regeneration[17,18,19]. Neutrophil accumulation was not significantly different in muscles of nondiabetic and diabetic mice (Fig. 3). On the other hand, macrophages showed significantly impaired accumulation in damaged muscle of both ob/ob and db/db mice, particularly at the 3-day time point. Similar to the regeneration data, WT mice fed the HFD did not show alterations in inflammatory cell accumulation compared to WT mice fed NC.

Diabetes has been associated with impaired neovascularization following ischemia, both in the heart and in the skeletal muscle[24,25]. Following cardiotoxin injury to the skeletal muscle, vascularity is disrupted early after injury as indicated by a decrease in CD31 labeling, which then increases above baseline values to a peak at 5 days postinjury (our unpublished data). As expected, a large number of CD31+ cells was observed in WT mice fed NC, indicating robust angiogenesis at 5 days postinjury (Fig. 4). In contrast, there was impaired CD31+ cell accumulation in muscles of both ob/ob and db/db mice (Fig. 4). WT mice fed the HFD showed no evidence of impaired angiogenesis.

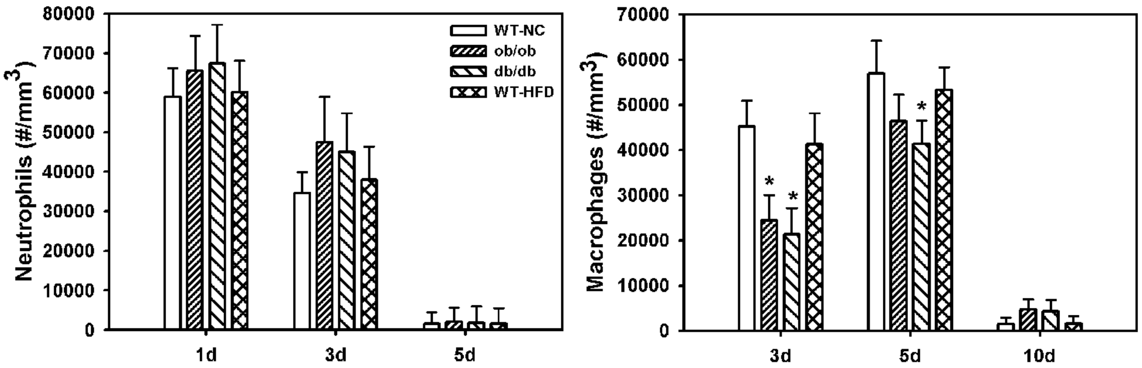

**FIGURE 3.** Neutrophil and macrophage accumulation following muscle injury. Injured muscles collected from WT mice fed NC, ob/ob mice, db/db mice, and WT mice fed HFD. Left: summary data for neutrophil accumulation assessed on days 1, 3, and 5 postinjury by immunostaining for the neutrophil-specific Ly6G antigen, counting number of Ly6G$^+$ cells per cryosection, and normalizing to cryosection volume. Right: summary data for macrophage accumulation assessed on days 3, 5, and 10 postinjury by immunostaining cryosections for the macrophage-specific F4/80 antigen, counting number of F4/80$^+$ cells per cryosection, and normalizing to cryosection volume. Bars are means ± standard error, n = 4–6 per time point. *= Mean value significantly smaller than that for WT-NC mice; $p < 0.05$.

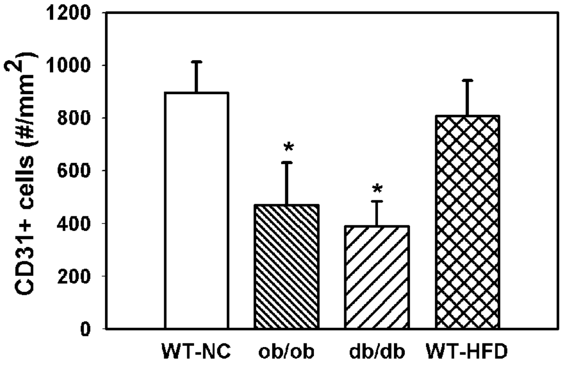

**FIGURE 4.** Angiogenesis following muscle injury is impaired in ob/ob and db/db mice. Injured muscles collected from WT mice fed NC, ob/ob mice, db/db mice, and WT mice fed HFD. Summary data for angiogenesis assessed on day 5 postinjury by immunostaining for the endothelial cell–specific CD31 antigen, counting number of CD31+ cells per cryosection, and normalizing to muscle area. Bars are means ± standard error, n = 4–6 per time point. *= Mean value significantly smaller than that for WT-NC mice; $p < 0.05$.

The impaired muscle regeneration in ob/ob and db/db mice could be the result of deficient leptin signaling and/or of downstream effects of dysregulated metabolic pathways. To begin assessing the potential role of leptin in muscle regeneration, the time course of leptin expression was measured in muscle following cardiotoxin-induced injury in WT mice fed NC and in a muscle cell line at different stages of differentiation (Fig. 5). Leptin expression was evident in undamaged muscle, and increased following injury with a peak at 5 days postinjury. In cultured muscle cells, leptin was expressed in both

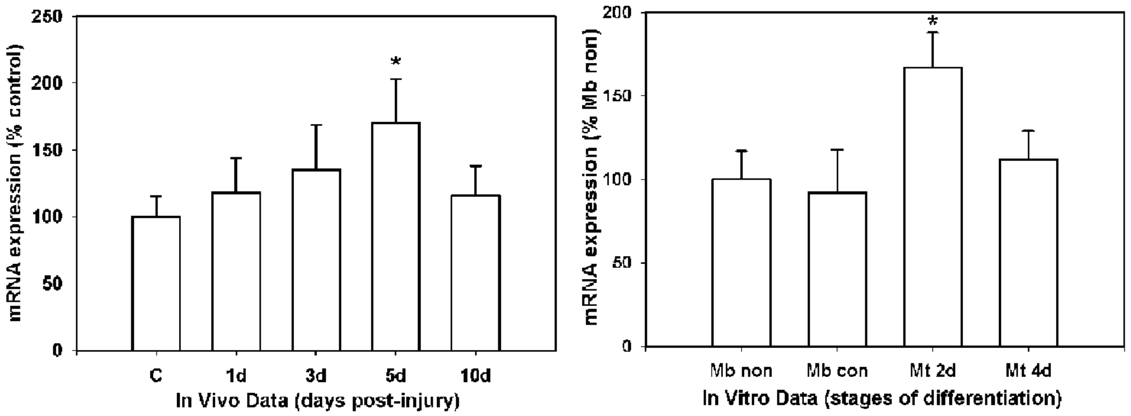

**FIGURE 5.** Leptin expression in skeletal muscle following injury and in muscle cells at different stages of differentiation. Leptin mRNA expression was evaluated by PCR, normalized to GAPDH expression, and displayed as percentage of uninjured control (left) or nonconfluent myoblast (right) values. Left: Leptin expression in uninjured control (C) muscle and muscles collected on days 1–10 postinjury. Right: Leptin expression in cultured C2C12 muscle cells at different stages of growth. Mb non: nonconfluent myoblasts; Mb con: confluent myoblasts; Mt 2d: myotubes 2 days after initiation of differentiation; Mt 4d: myotubes 4 days after initiation of differentiation. Bars are means ± standard error, n = 4 per time point. * = Mean value significantly larger than value for control muscles or nonconfluent myoblasts; $p < 0.05$.

proliferating and confluent myoblasts, but increased early during differentiation and then returned to baseline levels during later differentiation.

## DISCUSSION

Obesity and type 2 diabetes are major health problems in the U.S., with over 30% of all adults classified as obese and possessing an increased risk of type 2 diabetes, cardiovascular disease, and other chronic diseases[26]. Promoting healthy skeletal muscle function is an important component of therapeutic strategies for type 2 diabetes, because insulin resistance in the skeletal muscle is an early and significant event in the disease process[27]. Proper muscle function is also required for regular physical activity that is often prescribed for losing weight, improving insulin sensitivity, and reducing the risk of other chronic diseases associated with obesity and diabetes. The major findings of this study were that muscle regeneration was delayed in obese and type 2 diabetic ob/ob and db/db mice, but not in obese and insulin-resistant HFD-fed WT mice. Impaired muscle regeneration in ob/ob and db/db mice was associated with reduced macrophage accumulation, angiogenesis, cell proliferation, and myoblast activity. We speculate that impaired muscle regeneration in obese and diabetic patients could exacerbate already-compromised exercise capacity and insulin resistance.

In a previous study, muscle function was studied in uninjured and cardiotoxin-injured muscles in mouse models of type 1 diabetes, streptozocin-injected mice, and Akita mice[28]. Tibialis muscles showed reduced muscle mass, force production, and fatigue resistance both in uninjured and injured diabetic mice compared with nondiabetic mice. However, differences in inflammatory responses, myoblast activity, or morphological measurements of regeneration were not assessed. Thus, the functional impairments observed could not be linked to deficits in any specific phase of the regenerative process. In the present study, macrophage accumulation, myoblast activity, and muscle regeneration were impaired in ob/ob and db/db mice, which are models of type 2 diabetes. Whether similar deficits are observed in models of type 1 diabetes remains to be determined.

In the present study, the impaired muscle regeneration observed in ob/ob and db/db mice could have resulted directly from a loss of leptin signaling in cells involved in muscle regeneration or from downstream effects of the deficiency in leptin signaling (e.g., hyperglycemia, insulin resistance). Skeletal muscle cells express both leptin and its receptor, and leptin can activate the PI3K, ERK1/2, and p38 signaling pathways in muscle cells both *in vivo* and *in vitro*[29,30,31]. Our data indicate that leptin expression is increased in muscle following injury at the time when myoblast accumulation peaks, and myoblast differentiation and fusion is initiated. We also found that leptin is expressed in the C2C12 muscle cell line and increases early in the differentiation process of these cells. The latter data contrast with studies on human myoblasts in which leptin expression was reduced during differentiation[32]. In addition, exogenous leptin has been reported to stimulate porcine myoblast proliferation and inhibit differentiation[33], consistent with a role for leptin in signaling for the myoblast accumulation required for muscle regeneration. We are planning further studies in order to determine the relative importance of direct and downstream effects of leptin signaling on myoblast activity and muscle regeneration.

Although a review of the literature revealed no prior studies on muscle regeneration in ob/ob or db/db mice, insight may be gained from studies on skeletal muscle mass. Muscle mass is reduced in both ob/ob and db/db mice, both in terms of absolute measurements and those relative to total body mass[34,35,36,37,38]. These data indicate that leptin positively regulates muscle mass either directly or indirectly. Since glucocorticoids are increased in these mice and adrenalectomy partially restores muscle mass, glucocorticoids may contribute to decreased muscle mass by inhibiting protein synthesis and stimulating protein degradation[39,40]. However, adrenalectomy likely alters the levels of many different hormones and the specific role of glucocorticoids remains to be defined. Leptin may contribute directly to maintaining muscle mass since leptin stimulation of muscle cells activates the PI3K and ERK1/2 pathways, which have been implicated in muscle growth[29,30,31]. Leptin also up-regulates PPAR-α expression in muscle cells and increases fatty acid oxidation both *in vivo* and *in vitro*[41,42,43]. Whether these or other pathways contribute to muscle mass or muscle repair after injury has not been determined. In support of a direct role for leptin in regulating muscle mass, treatment of old mice with recombinant leptin for 10 days increased hindlimb muscle mass and muscle fiber area[44].

Leptin could also influence muscle regeneration through its effects on monocytes and macrophages. Our data are consistent with a role for leptin in macrophage function, as accumulation of mature macrophages was reduced in both ob/ob and db/db mice. Macrophages have been shown to be required for efficient muscle regeneration, as cell-specific deletion of macrophages results in decreased myoblast activity and impaired regeneration[17,18,19]. Monocytes and macrophages express leptin receptors and leptin appears to have multiple direct effects on these cells. Leptin promotes proliferation of isolated monocytes/macrophages, protects against apoptosis, and stimulates chemotaxis[45,46,47,48]. Leptin also enhances monocyte/macrophage activation through the classical or “proinflammatory” pathway, increasing expression of inflammatory molecules such as IL-12, TNF-α, and iNOS[46,47,48]. Interestingly, the proinflammatory macrophage phenotype appears to induce myoblast proliferation, whereas the alternative or noninflammatory macrophage phenotype appears to induce myoblast differentiation and fusion[17].

To our knowledge, our study provides the first data on muscle regeneration in ob/ob and db/db mice. However, leptin signaling is known to be required for the healing of injured skin. Healing is impaired in excisional skin wounds of both ob/ob and db/db mice and in wounds of WT mice treated with a leptin-blocking antibody[49,50,51]. Administration of topical exogenous leptin accelerated wound closure in WT mice and restored wound closure in ob/ob mice, but not db/db mice, indicating that leptin signaling contributes directly to wound healing[49,51]. Whether leptin signaling similarly regulates tissue repair following muscle injury remains unclear.

If leptin signaling is indeed critical for tissue repair, the loss of leptin sensitivity observed in obese and type 2 diabetic patients may predispose these patients to impaired healing. Such leptin resistance was initially observed in the central nervous system; leptin administration induces signaling in the hypothalamus of normal-weight mice, but such signaling is impaired in HFD-fed mice[52,53]. Similarly, leptin reduces food intake in normal mice, but not in HFD mice. Recently, the concept of leptin resistance has been extended to peripheral tissues. In muscle from normal-weight mice and humans, leptin stimulates skeletal muscle fatty acid oxidation, but this stimulation is impaired in muscle from obese individuals[42,54,55]. The mechanisms underlying leptin resistance remain to be elucidated, especially in peripheral tissues. In addition, whether leptin resistance plays a role in obesity-related impairments in muscle inflammation and repair has yet to be investigated.

In summary, muscle regeneration was impaired in obese and diabetic ob/ob and db/db mice, but not in WT mice made obese and insulin-resistant by HFD. Angiogenesis, cell proliferation, and myoblast accumulation were also impaired in ob/ob and db/db mice, but not in HFD mice. The impairments in muscle regeneration were associated with impaired accumulation of macrophages; which have been shown previously to be required for efficient muscle regeneration. Impaired regeneration in ob/ob and db/db mice could be due partly to the lack of leptin signaling, since leptin is expressed both in damaged muscle and in cultured muscle cells. These findings have implications for obese and diabetic patients for which impaired muscle regeneration following muscle injury caused by vigorous physical activity, peripheral ischemia, or trauma could exacerbate already compromised exercise capacity and insulin resistance

## ACKNOWLEDGMENTS

The authors thank Margaret Novak for her critical comments on a previous version of this manuscript.
